# Comparison of Current World Health Organization Guidelines with Physiologically Based Serum Ferritin Thresholds for Iron Deficiency in Healthy Young Children and Nonpregnant Women Using Data from the Third National Health and Nutrition Examination Survey

**DOI:** 10.1016/j.tjnut.2023.01.035

**Published:** 2023-02-02

**Authors:** Zuguo Mei, O Yaw Addo, Maria Elena D. Jefferds, Andrea J. Sharma, Rafael C. Flores-Ayala, Christine M. Pfeiffer, Gary M. Brittenham

**Affiliations:** 1 Division of Nutrition, Physical Activity, and Obesity, National Center for Chronic Disease Prevention and Health Promotion, United States Centers for Disease Control and Prevention, Atlanta GA, United States; 2 Division of Laboratory Sciences, National Center for Environmental Health, United States Centers for Disease Control and Prevention, Atlanta GA, United States; 3 Department of Pediatrics, College of Physicians and Surgeons, Columbia University, New York, NY, United States

**Keywords:** serum ferritin, hemoglobin, erythrocyte zinc protoporphyrin, iron deficiency, threshold, NHANES

## Abstract

**Background:**

Current WHO serum ferritin (SF) thresholds for iron deficiency (ID) in children (<12 μg/L) and women (<15 μg/L) are derived from expert opinion based on radiometric assays in use decades ago. Using a contemporary immunoturbidimetry assay, higher thresholds (children, <20 μg/L; women, <25 μg/L) were identified from physiologically based analyses.

**Objective:**

We examined relationships of SF measured using an immunoradiometric assay from the era of expert opinion with 2 independently measured indicators of ID, hemoglobin (Hb) and erythrocyte zinc protoporphyrin (eZnPP), using data from the Third National Health and Nutrition Examination Survey (NHANES III, 1988–1994). The SF at which circulating Hb begins to decrease and eZnPP begins to increase provides a physiological basis for identifying the onset of iron-deficient erythropoiesis.

**Methods:**

We analyzed NHANES III cross-sectional data from 2616 apparently healthy children, aged 12–59 mo, and 4639 apparently healthy nonpregnant women, aged 15–49 y. We used restricted cubic spline regression models to determine SF thresholds for ID.

**Results:**

SF thresholds identified by Hb and eZnPP did not differ significantly in children, 21.2 μg/L (95% confidence interval: 18.5, 26.5) and 18.7 μg/L (17.9, 19.7), and, in women, were similar although significantly different, 24.8 μg/L (23.4, 26.9) and 22.5 μg/L (21.7, 23.3).

**Conclusions:**

These NHANES results suggest that physiologically based SF thresholds are higher than the thresholds from expert opinion established during the same era. SF thresholds found using physiological indicators detect the onset of iron-deficient erythropoiesis, whereas the WHO thresholds identify a later, more severe stage of ID.

## Introduction

In 2020, the WHO published guidelines for the use of serum ferritin (SF) concentrations to assess iron status in apparently healthy individuals and populations [1]. After a comprehensive review of the available evidence, the WHO concluded that “available studies were not sufficient to justify a change in current ferritin cut-off values to define iron deficiency...” [1]. The WHO emphasized that these unchanged thresholds for SF (<12 μg/L for children and <15 μg/L for nonpregnant women) were not based on published data but were derived from qualitative expert opinion that was last revised in 1993 [2]. The 1993 SF thresholds were preserved during technical meetings for WHO guideline development in 2010, 2014, 2015, and 2016 [1]. In 2020, the WHO also cautioned that “Interpretation of and comparison between studies that have been undertaken in various laboratories at different times in the last decades may be compounded by variation and evolution in assay techniques and platforms, as well as the limited use of WHO reference materials.” [1]. Despite the availability of international reference materials, the standardization of immunoassays for SF is complicated by the differences in ferritin isoforms, antibodies, calibrators, assay methodologies, and the absence of physicochemical reference methods [3].

We adapted a method [4,5] for determining SF thresholds using physiological indicators of iron deficiency (ID) in conjunction with data from healthy populations. Using NHANES data from 2003 to 2006, 2007 to 2010, and 2015 to 2018, 2 independent indicators of iron-deficient erythropoiesis, hemoglobin (Hb) and soluble transferrin receptor (sTfR), identified SF concentrations of <20 μg/L for children and <25 μg/L for nonpregnant women [5], both are higher than the current WHO guideline [1]. The SF concentrations in the 2003–2008 NHANES were measured using an immunoturbidimetric assay developed in the decades since the formation of the expert opinion. The SF concentrations in the 2009–2010 and 2015–2018 NHANES were measured using an electrochemiluminescence assay, but we adjusted those data to match the earlier immunoturbidimetric assay [5]. In this article, we examined cross-sectional data from children and nonpregnant women participating in the Third NHANES (NHANES III, 1988–1994). NHANES III relied on an immunoradiometric SF assay that was widely used during that era but has since been discontinued.

NHANES III did not assess sTfR concentration but measured Hb and another indicator of iron-deficient erythropoiesis, erythrocyte zinc protoporphyrin (eZnPP). As body iron stores decline, SF decreases. With the onset of ID, iron needed for red cell production and tissue are not available to be mobilized from stores. A multitude of homeostatic mechanisms are then activated to maintain iron delivery to tissues by decreasing the iron used for erythropoiesis [5]. In the developing RBC, ID decreases the iron available for insertion into protoporphyrin IX, the final step in the synthesis of Hb. Divalent zinc is then incorporated instead, producing eZnPP, which persists for the life of the RBC as an indicator of ID. As iron deficits worsen, the circulating Hb concentration falls. Consequently, the SF concentration at which the circulating Hb concentration begins to decrease and the eZnPP concentration begin to increase, provides another physiological basis for detecting the onset of ID erythropoiesis.

The aim of our study was to compare SF thresholds for ID between the 2020 WHO guidelines and newly developed physiologically based determinations for healthy children and nonpregnant women [5]. We compared the current WHO SF thresholds for ID, derived from expert opinion last revised in 1993, with the NHANES III (1988–1994) derived thresholds wherein SF results were generated with an immunoradiometric assay from that period. In addition, to more fully characterize the effects of ID on circulating RBCs, we examined the distributions of SF with RBC measures in the NHANES III data, including the mean corpuscular volume (MCV), mean corpuscular hemoglobin (MCH), and RBC distribution width (RDW). We identified an apparently healthy subsample of children and nonpregnant women in NHANES III data, while noting that this subsample cannot be used to make nationally representative estimates for the healthy or general US population. We examined the distributions of SF, Hb, and eZnPP concentrations and then used restricted cubic spline (RCS) analysis to identify the SF threshold corresponding to the onset of iron-deficient erythropoiesis.

## Methods

### Study population and sample selection

Data from NHANES III, 1988–1994 represent the civilian, noninstitutionalized population in the United States in that period. NHANES is a multi-purpose survey designed to assess the health and nutritional status of adults and children in the United States that includes an interview in the household followed by a standardized health examination in a mobile examination center (MEC) [6–8]. NHANES III relies on a stratified multistage probability sample based on the selection of counties, blocks, households, and persons within households. The surveys were conducted by the *National Center for Health Statistics* at the *CDC*. Ethical approval was obtained and written informed consent was obtained from participants aged ≥12 y. Parental consent was obtained for those aged <18 y. Procedures for data collection and analysis are published elsewhere [6–8].

**FIGURE 1.**
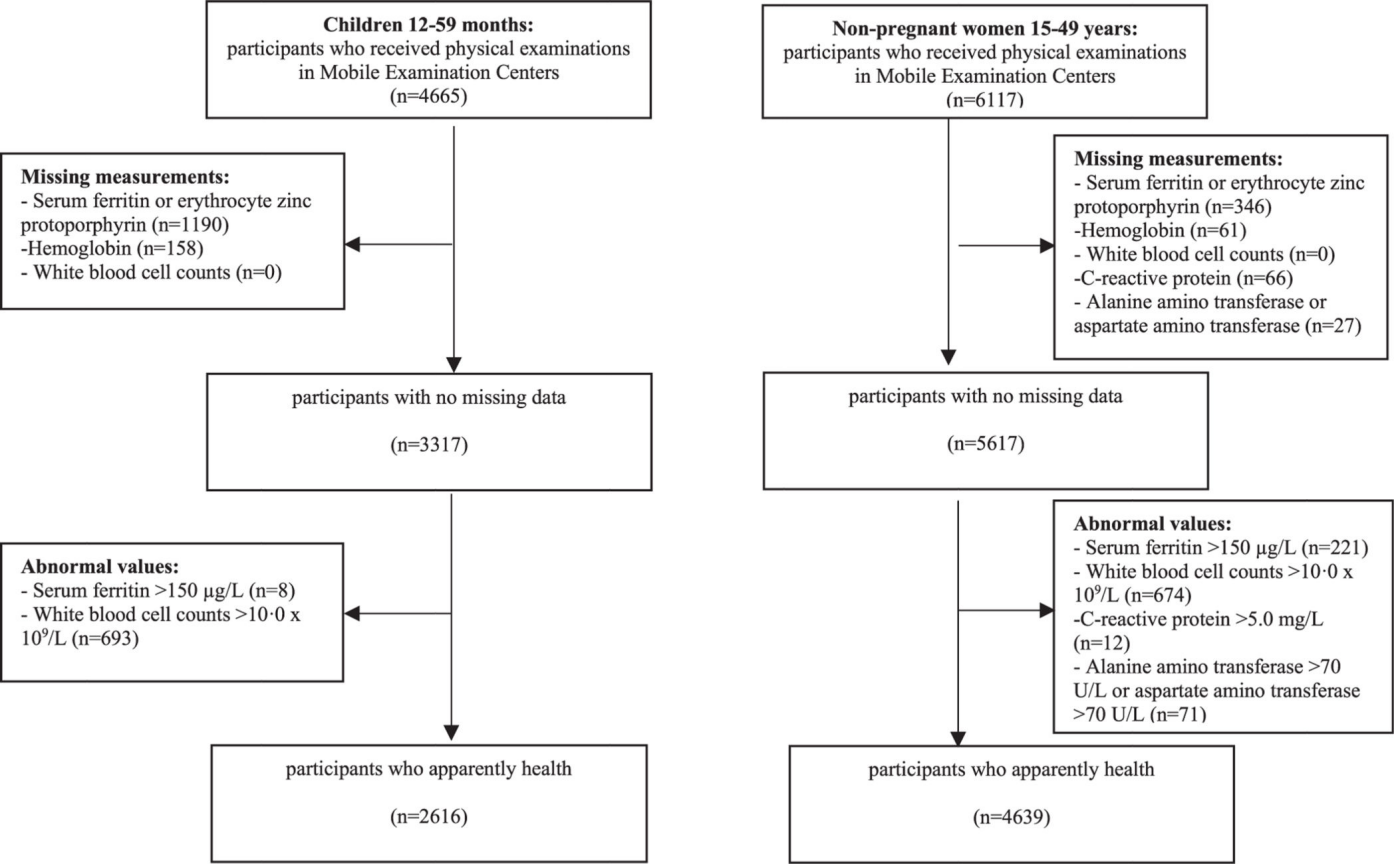
Flowchart of selection of healthy sample of US children aged 12–59 mo and nonpregnant women aged 15–49 y participating in the Third National Health and Nutrition Examination Survey 1988–1994.

NHANES III measured SF, Hb, MCV, MCH, RDW, and eZnPP [9]. For the main analysis, we restricted our study sample to participants who received health examinations in MEC; 4665 children aged 12–59 mo and 6117 nonpregnant women aged 15–49 y. We then identified an apparently healthy subsample by using NHANES III available data to exclude individuals with other common causes of anemia at the population level that are independent of ID, namely children or nonpregnant women with an indicator of infection (white blood cell counts >10.0 × 10^9^/L) or at risk of iron overload (current WHO cut-off for severe risk of iron overload is SF >150 μg/L for females of all ages >5 y [1]). In addition, nonpregnant women with inflammation (CRP >5.0 mg/L [10]), or potential liver disease (as defined by abnormal elevations of ALT >70 U/L or AST >70 U/L [11]). Our final sample included 2616 children and 4639 nonpregnant women. Detailed sample selection for both children and nonpregnant women are shown in Figure 1. For children, this represents 56.1% of the originally examined sample. For nonpregnant women, this represents 75.8% of the examined sample. Participants excluded because of missing SF did not differ from those included by race or ethnicity or sex distributions (among children), but they differed by age. Children and females, aged 15–19 y, were more likely than older participants to be excluded owing to missing SF results (Chi-square test, *P* < 0.01). The amount of serum collected is smallest for the youngest age group (12–23 mo) and increases with age group up to age ≥12 y. Owing to assay prioritization, participants with smaller specimen volume will be more likely to miss a lower priority assay for NHANES III, such as SF. This could explain the greater percentage of missing SF results among children aged 12–59 mo.

For the additional analysis of the distributions of SF with RBC measures in the NHANES III data (MCV, MCH, and RDW), we used the same criteria mentioned above to define healthy children and nonpregnant women with nonmissing SF, MCV, MCH, and RDW. The final sample included 2637 children and 4648 nonpregnant women.

### Laboratory analysis

NHANES III measured SF, eZnPP, Hb, MCV, MCH, RDW, CRP, ALT, and AST [9]. SF, Hb, MCV, MCH, RDW, and eZnPP were measured for both children aged 12–59 mo and nonpregnant women aged 15–49 y, but CRP was measured only for participants aged ≥4 y. ALT and AST were measured only for participants aged ≥12 y [8]. Both SF and eZnPP were measured in the NHANES Laboratory, National Center for Environmental Health at the CDC.

SF was measured using the BioRad Laboratories “QuantImune Ferritin IRMA” kit [12], which is a single-incubation 2-site immunoradiometric assay (IRMA). In this IRMA, which measured the most basic isoferritins, the highly purified ^125^I-labeled antibody to ferritin was the tracer, and the ferritin antibodies were immobilized on polyacrylamide beads as the solid phase [9].

eZnPP was measured by a modification of the method of Sassa et al. [13] using a Hitachi Model F-2000 fluorescence spectrophotometer [9]. Protoporphyrin was extracted from EDTA-whole blood into a 2:1 (v/v) mixture of ethyl acetate-acetic acid, then back-extracted into diluted hydrochloric acid. The protoporphyrin in the aqueous phase was measured fluorometrically at excitation and emission wavelengths of 404 and 655 nm, respectively. Calculations were based on a processed protoporphyrin IX (free acid) standard curve. After a correction for the individual hematocrit was made, the final concentration of eZnPP in a specimen was expressed as micrograms per deciliter of packed RBCs (μg/dL RBC) [9]. ID was defined as eZnPP >1.42μmol/L for children aged 12–23 mo, >1.24 μmol/L for 24–59 mo, and >1.24 μmol/L for all nonpregnant women [14].

Hb, MCV, MCH, and RDW were part of the hematology parameters in whole blood that were measured as part of a complete blood count in the MEC using the *Coulter Counter Model S-PLUS JR* with Coulter histogram differential, hereafter referred to as the Model S-PLUS JR, is a quantitative, automated hematology analyzer [9]. Anemia was defined as Hb <11.0 g/dL for children and <12.0 g/dL for nonpregnant women [15]. Complete blood counts, including white blood cell counts, were also measured in the MEC using the *Coulter Counter Model S-PLUS JR* [9]. We used the original Hb data without adjustment for altitude (data unavailable) or smoking status in nonpregnant women.

CRP was measured using serum at the *University of Washington* (Seattle, WA) by latex-enhanced nephelometry (Behring Nephelometer Analyzer System, Behring Diagnostics Inc., Somerville, NJ) [7]. Both ALT and AST were part of the standard biochemistry profile that was measured using serum in the *White Sands Research Center* (Alamogordo, NM), using a Hitachi Model 737 multichannel analyzer (Boehringer Mannheim Diagnostics, Indianapolis, IN) [9].

### Statistical analysis

First, we log-transformed SF [log_10_(SF)] data to normalize the distributions, as previous studies suggested that the SF distribution is a typically right-skewed distribution [1], then calculated geometric means and 95% CIs and described the SF distributions with basic characteristics for children aged 12–59 mo and nonpregnant women aged 15–49 y. For basic characteristics and SF distribution analysis, we used SUDAAN (version 11.0.1; Research Triangle Institute, Research Triangle Park, NC) with examination sample weights and design variables to account for the complex sample design. A Bonferroni adjustment [16] was used to correct the *P* values for the significance test for the multiple comparisons across each socio-demographic characteristic in SF.

Using exploratory data analysis techniques [17], we examined monotonic relationships between Hb and SF, and between eZnPP and SF using a scattergram superimposed with a plot of concentrations of median Hb and median eZnPP according to the categories of SF levels for nonpregnant women (1 μg/L), and for children, (5 μg/L, with the larger category range because of the smaller sample size). RCS regression models with 5 knots [18, 19] were used to examine the relationship of: *1)* continuous SF with Hb; and *2)* continuous SF with eZnPP for both children and nonpregnant women to identify the potential SF thresholds for ID. We used 2 complementary analyses to achieve our objectives. First, with Hb as our outcome variable, we modeled its relationship with SF and then solved for the SF concentration corresponding to the Hb plateau. Second, to further ascertain the consistency of this derived SF threshold, we then modeled SF against eZnPP and solved for the SF value corresponding to the eZnPP minima. Using the fitted function of these 2 separate RCS, we solved the ordinary differential equation derivative solutions at the plateau or minimum of each model, with both in SF units. The above RCS analysis methods were also repeated after stratification of the NHANES III data by age groups for children and nonpregnant women to examine variation in age groups.

For the RCS analysis, R software 4. 2.1 (R Foundation for Statistical Computing and Graphics, Vienna, Austria) was used without accounting for the complex sample design because we did not intend to generate nationally representative results using the RCS analysis but rather to understand the biologic relationships within the sampled individuals with respect to these iron status indicators. Bootstrap resampling techniques were used to generate the 95% CI around each plateau or minima point estimate derived from the differential solution of each RCS fit. For each model, 5000 replications were generated, and the 95% Cis estimates were corrected for bias using bias corrected acceleration [20]. Cochrane’s Q test for heterogeneity in the calculated SF thresholds were performed using 2-sided random effect meta-analysis [21]. Statistical significance was set at *a priori* and 2-tailed *P* < 0.05 for all analyses.

## Results

Geometric mean SF concentrations were 23.1 μg/L (95% CI: 22.3, 23.8) for children 12–59 mo and 30.2 μg/L (95% CI: 28.8, 31.8) for nonpregnant women 15–49 y, respectively (Table 1), and the difference was statistically significant (2-tailed t-test, *P* < 0.001). The lower 5th percentiles were 6.9 μg/L for children and 5.5 μg/L for nonpregnant women. In children, geometric mean SF concentrations were higher in girls than in boys and were higher among older children (24–59 mo) than younger children (12–23 mo). Non-Hispanic Black children had higher SF concentrations than either non-Hispanic White or Mexican-American children (Table 1). SF concentrations differed by age group in nonpregnant women. Nonpregnant women aged 20–49 y had higher SF concentrations than adolescents 15–19 y (Table 1). Non-Hispanic white nonpregnant women had higher SF concentrations than non-Hispanic black or Mexican-American women (Table 1).

**FIGURE 2.**
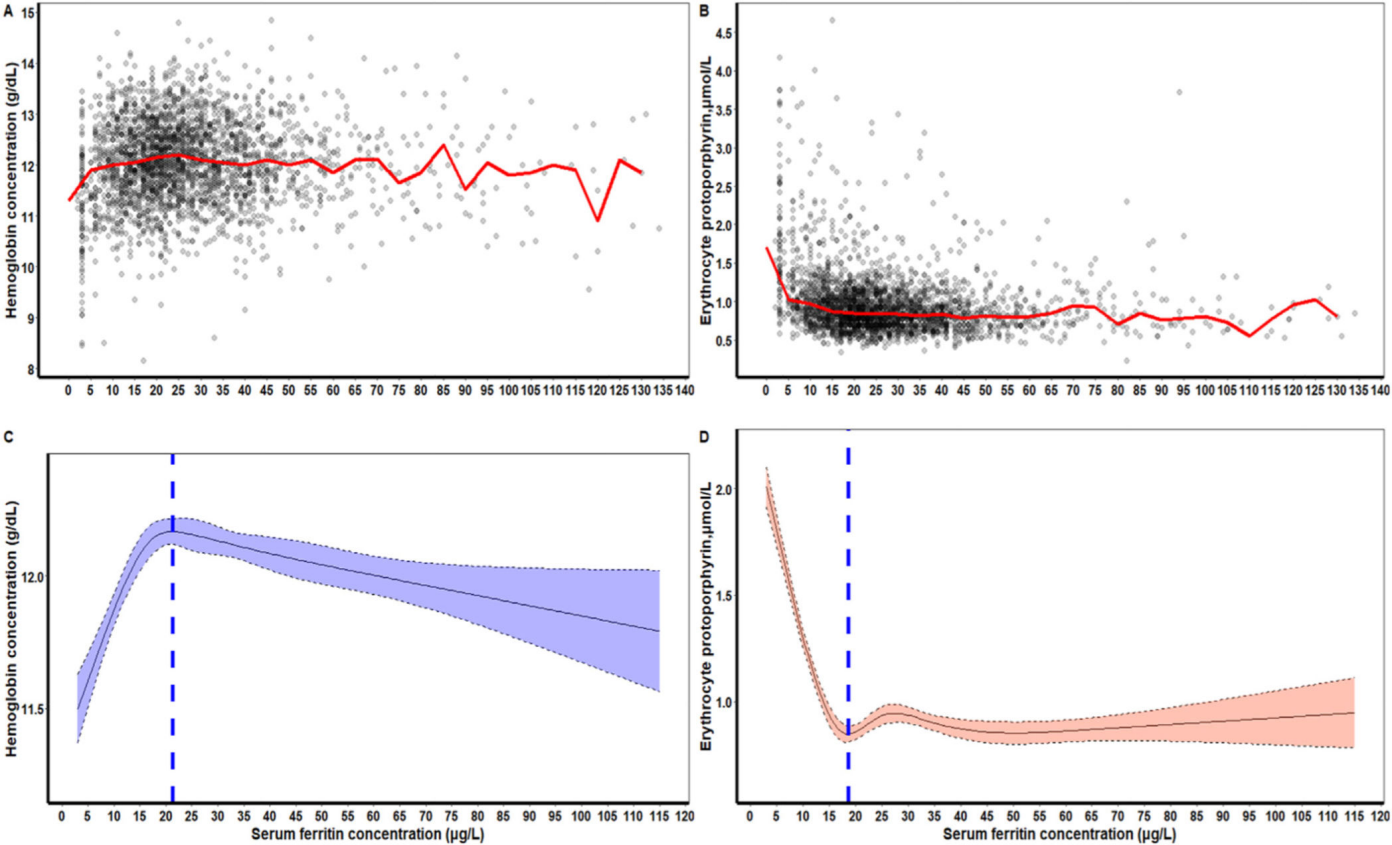
(A) Plot of SF (μg/L) concentrations with median hemoglobin (Hb, g/dL, red line), and (B) median erythrocyte zinc protoporphyrin (eZnPP, μmol/L, red line), and (C) the restricted cubic spline (RCS) regression with 5 knots (Hb, vertical blue line indicates the plateau point; (D), eZnPP vertical blue line indicates the minima point. Shaded areas inside the dashed lines represent 95% CIs), in a healthy sample of United States children aged 12–59 mo (*n* = 2616) participating in the Third National Health and Nutrition Examination Survey 1988–1994 (the following exclusion applies to define a healthy sample: children with infection).

**FIGURE 3.**
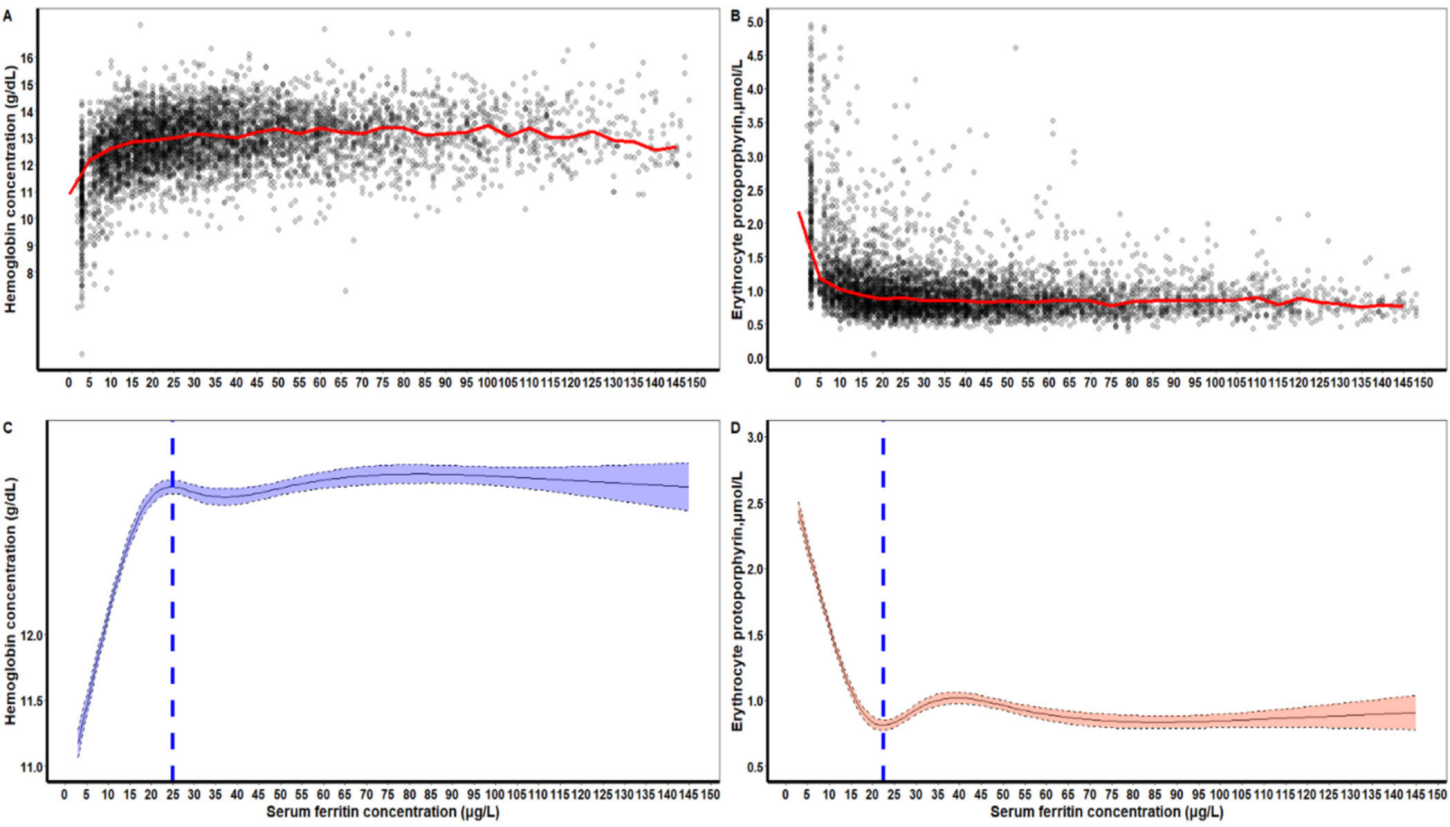
(A) Plot of SF (μg/L) concentrations with median hemoglobin (Hb, g/dL, red line) and (B) median erythrocyte zinc protoporphyrin (eZnPP, μmol/L, red line), and (C) the restricted cubic spline (RCS) regression with 5 knots (Hb, vertical blue line indicates the plateau point; Panel D, eZnPP, vertical blue line indicates the minima point. Shaded areas inside the dashed lines represent 95% CIs), in a healthy sample of United States nonpregnant women aged 15–49 y (*n* 4639) participating in the Third National Health and Nutrition Examination Survey 1988–1994 (the following exclusion applies to define a healthy sample: nonpregnant women with infection, inflammation or liver disease).

Figure 2 panel A and B were the plots of the median Hb to SF and eZnPP to SF, respectively, in children. Figure 3 panel A and B were the plots of the medium Hb to SF and eZnPP to SF, respectively, in nonpregnant women. These empirical distribution plots indicated clear nonlinear trends with distinct thresholds conspicuous at certain levels of SF (*x-axis*), and inflection points at levels of Hb or eZnPP (*y-axis*). Using RCS regression analysis, we found curvilinear relationships between SF and median Hb and between SF and median eZnPP for both children and nonpregnant women. In children, the SF values corresponding to both the Hb plateau point and the eZnPP minima point were 21.2 μg/L and 18.7 μg/L, respectively that did not differ significantly (Table 2). The RCS regression curves for both median Hb and median eZnPP are presented in Figure 2C and D. When stratified by age, the corresponding plateau points in older children (24–59 mo) was significantly higher than younger children (12–23 mo) for median Hb but not for median eZnPP (Supplemental Table 1).

The patterns in nonpregnant women were generally similar to those found in children (Table 2). The SF values corresponding to the median Hb plateau point and to the median eZnPP minima point differed significantly: 24.8 μg/L for median Hb and 22.5 μg/L for median eZnPP, respectively (Table 2). The RCS regression curves for both median Hb and median eZnPP were presented in Figure 3C and D. The corresponding plateau point in older nonpregnant women (20–49 y) also differed significantly from that in younger nonpregnant women (15–19 y) for both Hb and eZnPP (Supplemental Table 2).

**FIGURE 4.**
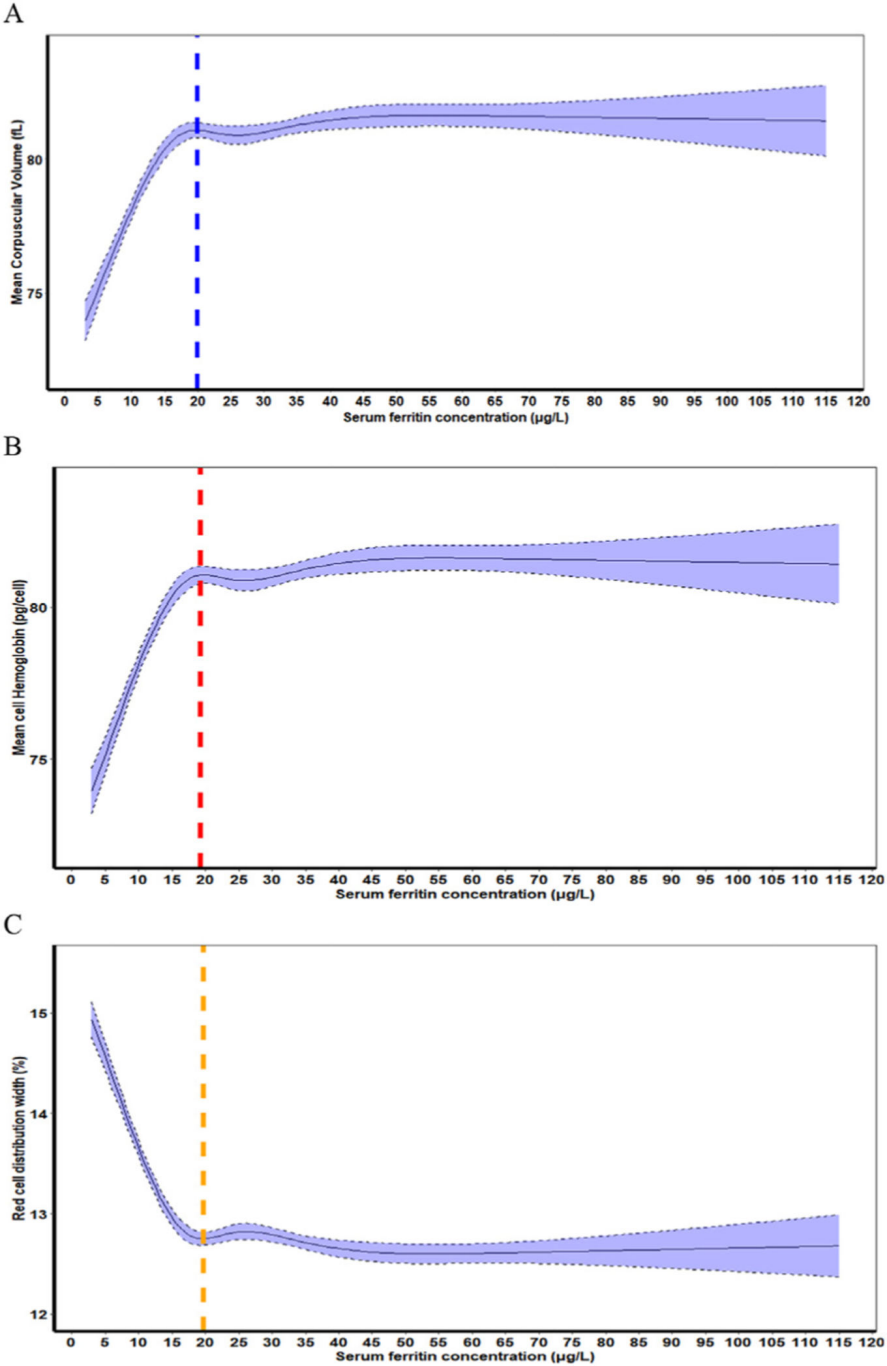
(A) Plot of the restricted cubic spline (RCS) regression with 5 knots of SF (μg/L) concentrations with mean corpuscular volume (MCV), vertical blue line indicates the plateau point), (B) MCH, vertical red line indicates the plateau point), and (C) RBC distribution width RDW, vertical yellow line indicates the plateau point). Shaded areas inside the dashed lines represent 95% CIs), in a healthy sample of United States children aged 12–59 mo (*n* = 2637) participating in the Third National Health and Nutrition Examination Survey 1988–1994 (the following exclusion applies to define a healthy sample: children with infection).

**FIGURE 5.**
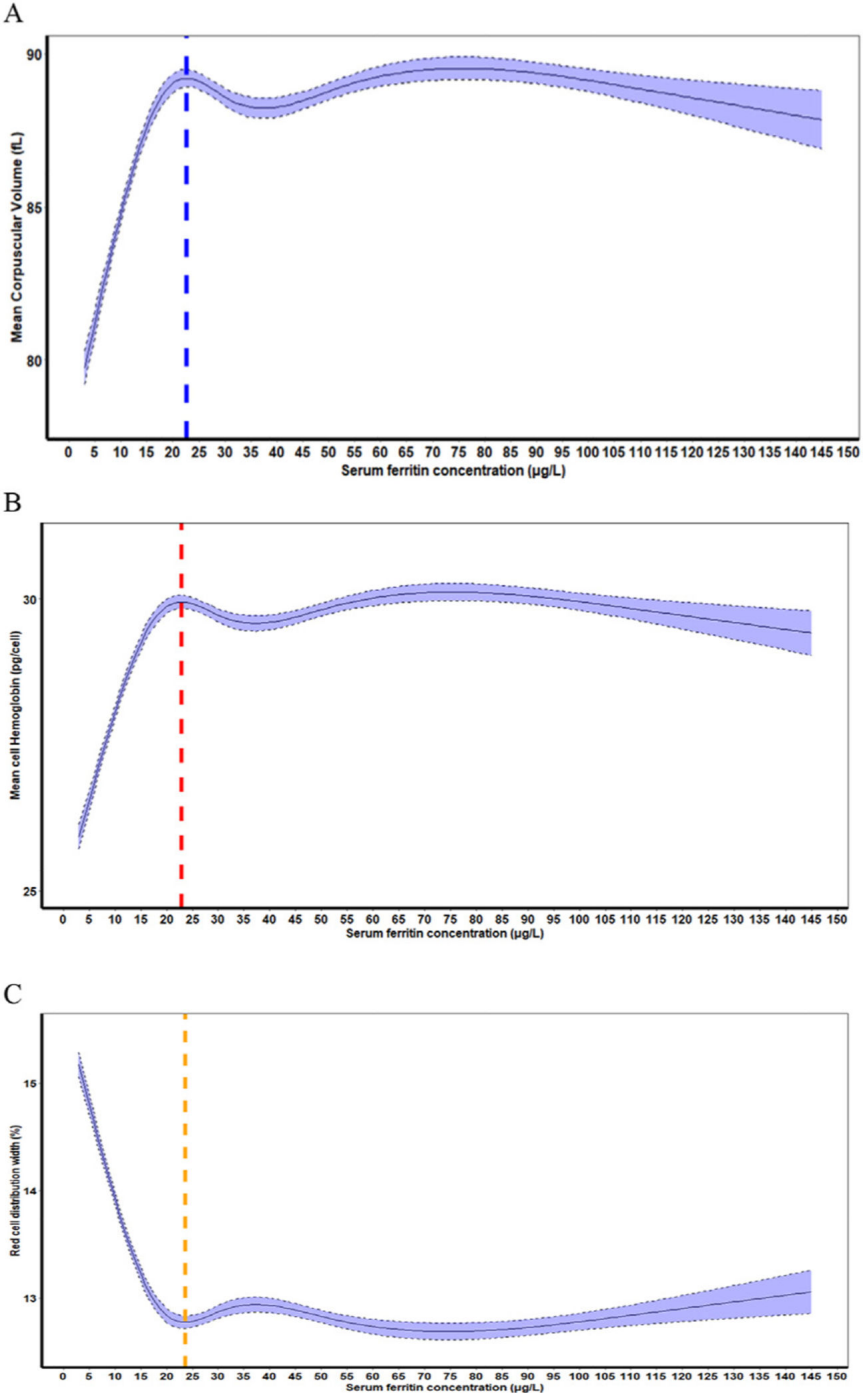
(A) Plot of the restricted cubic spline (RCS) regression with 5 knots of SF (μg/L) concentrations with mean corpuscular volume (MCV), vertical blue line indicates the plateau point), (B) MCH, vertical red line indicates the plateau point), and (C) RBC distribution width (RDW, vertical yellow line indicates the plateau point). Shaded areas inside the dashed lines represent 95% CIs), in a healthy sample of United States nonpregnant women aged 15–49 y (*n* = 4648) participating in the Third National Health and Nutrition Examination Survey 1988–1994 (the following exclusion applies to define a healthy sample: nonpregnant women with infection, inflammation, or liver disease).

Tables 3 presents analyses of the distributions of SF with RBC indices (MCV, MCH, and RDW). In children, the SF values corresponding to the median MCV and MCH plateau points, or RDW minima point were: 19.9 μg/L, 19.2 μg/L, and 19.7 μg/L, respectively (Table 3). In nonpregnant women, the SF concentrations corresponding to the median MCV and MCH plateau points, or RDW minima point were: 22.7 μg/L, 22.9 μg/L, and 23.6 μg/L, respectively (Table 3). Moreover, for both the groups, they did not differ significantly (Table 3). The RCS regression curves for median MCV, MCH, and RDW are presented in Figure 4 for children and Figure 5 for nonpregnant women.

## Discussion

**FIGURE 6.**
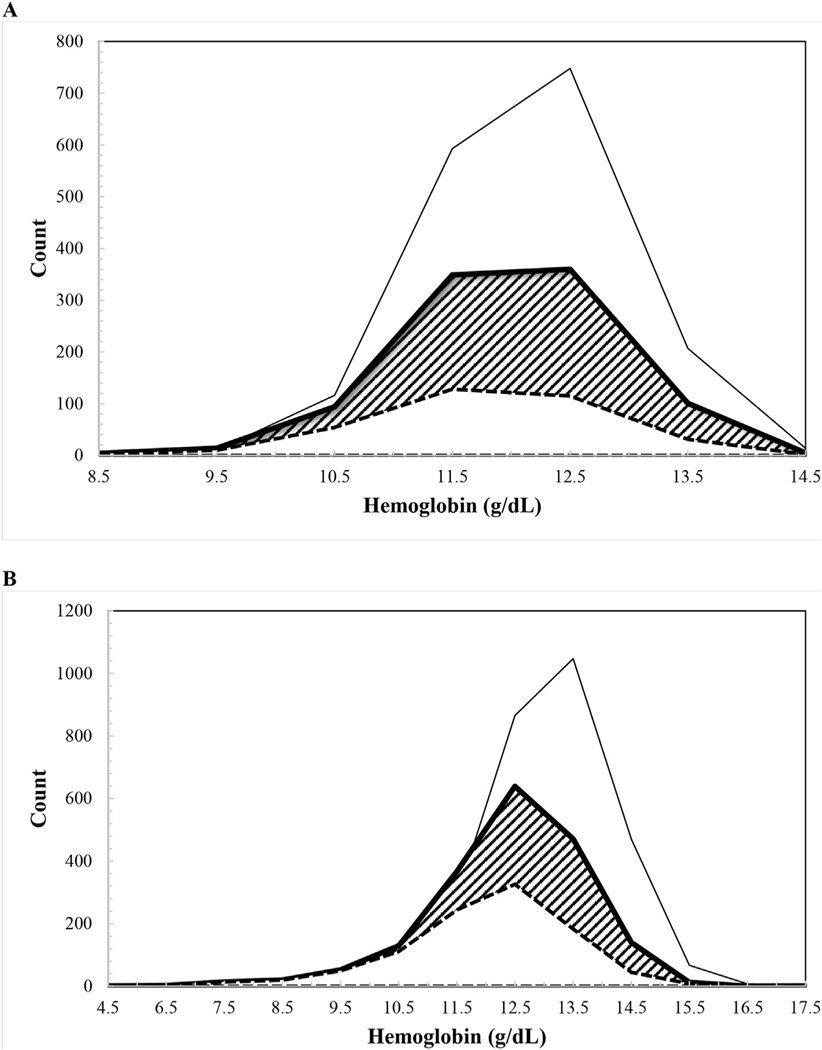
Hemoglobin distribution in children by serum ferritin (SF) concentration groups [(A), SF<12 μg/L (*n* = 343, dashed line), SF<20 μg/L (*n* = 927, thick line), and SF≥20 μg/L (*n* = 1689, thin line)] and in nonpregnant women [(B) SF<15 μg/L (n = 1001, dashed line), SF<25 μg/L (*n* = 1847, thick line), and SF≥25 μg/L (*n* = 2792, thin line)], in a healthy sample of United States children aged 12–59 mo (*n* = 2616) and nonpregnant women aged 15–49 y (*n* = 4639) participating in the Third National Health and Nutrition Examination Survey 1988–1994. The shaded areas identify the additional individuals identified as iron-deficient using the physiologically based concentrations compared to the WHO guidelines (*n* = 584, children. *N* = 846, nonpregnant women) corresponding to an additional 22.2% and 17.3% children and nonpregnant women, respectively.

Our analyses provide physiological evidence from NHANES III, 1988–1994, that iron-deficient erythropoiesis began at SF concentrations of 20 and 25 μg/L in children and nonpregnant women, respectively, measured by an immunoradiometric SF assay widely used in that period (Table 2; Figures 2 and 3). These SF thresholds in children and nonpregnant women were higher than the WHO thresholds of <12 (children) and <15 μg/L (nonpregnant women) [1], which were last revised during a 1993 expert consultation. Using the physiologic approach, our earlier study [5] using data from NHANES 2003–2006, 2007–2010, and 2015–2018 also determined higher SF thresholds than the WHO expert opinion-based thresholds [1]. The SF assays used to generate the latter NHANES data measured somewhat higher than the BioRad assay used earlier in NHANES, closer to the time when the WHO expert opinion-based thresholds were formulated, which could have contributed to the high SF thresholds. Our current study, therefore, utilized the older NHANES data generated with the BioRad assay to assess whether SF thresholds determined using the physiologic approach were again higher than the WHO expert opinion-based thresholds. Not only did we confirm the high thresholds, but the differences between the current WHO guideline and physiologically based SF concentrations are virtually identical to those from our previous analysis [5], as well as those for healthy nonpregnant women who are blood donors [22]. Physiologically based SF concentrations are potentially important, both clinically and epidemiologically, for identifying increased proportions of individuals and populations with ID. In the US NHANES III apparently healthy populations, the higher physiologically based SF concentrations would recognize more children and nonpregnant women, respectively, as iron-deficient (shaded areas in Figure 6, A and B). The numbers shown in Figure 6 correspond to an additional 22.2% and 17.3% children and nonpregnant women, respectively. Higher SF thresholds could help assure that earlier ID is detected and help allow clinical management and public health interventions to avoid progression to more severe iron deficits and anemia.

The physiologically identified SF concentrations in NHANES III are for nutritional ID, considered the primary cause of iron deficits in the US population. Nutritional ID is the result of a sustained imbalance between the intake and absorption of bioavailable iron and iron requirements. The progression of the development of nutritional ID has long been characterized by effects on the circulating Hb concentration [23]. *Iron depletion* develops when stores are exhausted, but the iron supply is maintained for red cell production and tissue requirements; the Hb concentration is unaffected. *Iron-deficient erythropoiesis*, also known as ID without anemia, develops when the Hb concentration begins to fall as a result of a failure of the iron supply for RBC production and tissue needs; the Hb concentration is maintained above the criterion used to define anemia. *Iron deficiency anemia* is identified when the insufficient supply of iron for erythropoiesis and tissue causes the Hb to fall below the standard for anemia. This gradual progression through successive stages is present when ID is solely the result of an inadequate iron intake or absorption.

A multitude of mechanisms have evolved to preserve iron delivery to nonerythroid tissues for essential metabolic functions. As iron stores become depleted, absorption increases [24]. In recent years, a variety of adaptations of developing and circulating RBCs to maintain the iron supply to tissue have been recognized [25]. During the early stages of erythroid differentiation, large amounts of iron are accumulated in preparation for Hb synthesis. If the iron supply falls, then export of iron from both developing and circulating RBCs to plasma helps protect iron delivery to nonerythroid cells and tissues [25]. Iron regulation of RBC differentiation helps match the rate of erythropoiesis to iron supply. Both heme and globin synthesis are coordinated with iron availability [26]. As ID develops, zinc replaces iron during the final step of Hb synthesis, forming eZnPP [27], which is than retained throughout the life span of the RBC. The effects of ID are not restricted to the Hb concentration alone and affect each manufactured RBC [26]. As the iron deficit becomes progressively more severe, the RBCs produced contain less and less Hb (decreased MCH, hypochromic), and become smaller and smaller (decreased MCV, microcytic), and the dispersion in red cell volumes increases (increased RDW). Because the red cell lifespan is around 90–120 d, approximately 1% of the circulating RBCs are replaced each day. When the newly produced hypochromic microcytic RBCs decrease the average circulating Hb concentration, iron-deficient erythropoiesis is identified. As shown in Table 3 and Figure 4, these changes in circulating RBCs occur in synchrony at SF concentrations well above the WHO thresholds [1]. Moreover, relative to the distribution of circulating Hb in the apparently healthy iron-replete populations of children and nonpregnant women, the distributions are shifted toward lower Hb concentrations to a greater extent using WHO SF thresholds than with the physiologically based SF values (Figure 5). Altogether, these results indicate that the physiologically identified SF concentrations in NHANES III detect an earlier, less severe stage of treatable ID than the current WHO guideline. Detecting individuals at an earlier stage of ID predominantly results in identifying a larger proportion of individuals as iron-deficient both with and without anemia.

Both the WHO in 2020 [1] and a recent update of the related Cochrane systematic review [28] evaluated the overall certainty of evidence for the SF threshold recommendation for iron deficiency as “low to very low.” The reference standard for ID was absence of iron stores as determined using a Perl’s (Prussian blue) stain on aspirated bone marrow examination. Only 3 eligible studies including 296 adult women (*N* = 203, Hallberg et al. [29], 1993; *N* = 29, Milman et al. [30], 1983; *N* = 64, Sorbie et al. [31], 1975), and no studies in young children met criteria for inclusion. The Cochrane review concluded that the “results for iron deficiency do not adequately indicate the most appropriate thresholds” but that a “ferritin threshold below 15 to 30 μg/L appears to indicate absent bone marrow iron stores in healthy populations” [19]. The physiologically based analysis of NHANES data have resulted in identification of SF thresholds that are compatible with this conclusion by using high-quality data that are based on carefully controlled laboratory analyses. An alternative possibility is that a small quantity of storage iron may be needed for an efficient delivery of iron from recycling macrophages to developing RBCs and nonerythroid tissues so that the onset of iron-deficient erythropoiesis precedes the absolute absence of storage iron [23].

Our age-stratified thresholds for SF in children were significantly different with Hb but not with eZnPP (Supplemental Table 1). In nonpregnant women, however, SF thresholds were significantly higher with both Hb and eZnPP in those aged 20–49 than those aged 15–19 y (Supplemental Table 2). Future studies should focus on whether age-stratified thresholds are warranted in children and nonpregnant women.

Our study has limitations. Although the immunoradiometric SF assay used in NHANES III was widely used at the time, its representativeness of the SF assays in use during the era of the formation of expert opinion is uncertain. The methods used to identify an apparently healthy population may have failed to exclude all those with inflammation, infection, or liver disease, and those with other causes of anemia or with conditions that increase SF independently of body iron stores. The individuals included were limited to the US population; thus, the use of international data from other healthy populations is needed to assess the generalizability and validity of the physiologically based analysis.

Although recognizing these and other potential limitations, the similarity of the potential SF thresholds identified in data from NHANES III, 1988–1994, with those from NHANES 2003–2006, 2007–2010, and 2015–2018 supports the reproducibility of the physiologically based approach. Overall, the use of population data to derive thresholds may be more clinically and epidemiologically applicable than reliance on results from expert opinion or the small number of healthy individuals who have undergone bone marrow examination.

## Supplementary Material

Supplemental material

## Figures and Tables

**TABLE 1:** Geometric means, medians, and selected percentiles (95% CI in parentheses) for SF (μg/L) concentrations in a healthy population^1^ of United Sates children aged 12–59 mo and nonpregnant women 15–49 y participating in the Third National Health and Nutrition Examination Survey (1988–1994)

	*n*	Geometric mean^2^	Median	5th percentile	95th percentile
Children 12–59 mo					
Total	2616	23.1 (22.3, 23.8)	23.6 (22.7, 25.1)	6.9 (6.0, 8.8)	60.6 (56.2, 67.2)
Age					
12–23 mo	494	18.1 (16.3, 20.0)^a^	19.7 (18.1, 21.9)	2.9 (2.6, 5.2)	55.7 (43.6, 60.4)
24–59 mo	2122	24.4 (23.4, 25.3)^b^	24.3 (23.4, 25.9)	8.9 (8.0, 10.4)	62.8 (55.7, 69.9)
Sex					
Male	1309	21.7 (20.7, 22.7)^a^	22.1 (21.0, 23.4)	6.5 (5.5, 8.2)	58.1 (50.6, 65.6)
Female	1307	24.7 (23.5, 25.9)^b^	25.2 (24.0, 26.6)	7.5 (6.5, 9.2)	63.6 (54.7, 70.7)
Race/ethnic group					
Non-Hispanic white	727	23.2 (22.1, 24.2)^a^	23.0 (21.6, 24.1)	8.2 (6.6, 10.0)	60.3 (50.3, 68.5)
Non-Hispanic black	887	25.9 (24.4, 27.4)^b^	27.1 (25.6, 28.7)	7.1 (5.6, 9.4)	63.6 (58.0, 69.9)
Mexican-American	857	19.4 (18.0, 20.9)^c^	20.4 (19.0, 22.1)	2.8 (2.6, 3.5)	60.6 (51.0, 66.6)
Nonpregnant women 15–49y					
Total	4639	30.2 (28.8, 31.8)	32.9 (31.3, 35.5)	5.5 (5.1, 6.8)	103 (98.2, 108.4)
Age					
15–19 y	810	24.7 (22.9, 26.7)^a^	26.1 (23.7, 27.9)	5.8 (5.1, 7.0)	82.6 (67.0, 94.4)
20–34 y	2138	32.2 (30.2, 34.4)^b^	35.5 (32.3, 40.0)	6.2 (5.7, 8.8)	98.3 (95.1, 105)
35–49 y	1691	30.1 (27.9, 32.4)^b^	33.4 (31.2, 37.8)	4.9 (3.9, 6.3)	111 (104, 117)
Race/ethnic group					
Non-Hispanic white	1435	32.2 (30.4, 34.1)^a^	34.9 (33.2, 38.4)	6.4 (5.9, 8.1)	101 (95.8, 108)
Non-Hispanic black	1586	29.4 (28.3, 30.6)^b^	32.1 (30.4, 34.8)	4.3 (3.7, 6.1)	116 (113, 119)
Mexican-American	1401	24.0 (22.9, 25.1)^c^	25.3 (23.8, 27.8)	2.7 (2.6, –^3^)	100 (93.9, 108)

1 Unweighted n, all other analyses are weighted. The following exclusions apply to define a healthy sample: infection (white blood cell counts >10.0 × 10^9^/L, all ages), inflammation (CRP >5.0 mg/L, participants aged ≥15 y), potential liver disease (alanine AST >70 U/L or aspartate AST >70 U/L, participants aged ≥15 y), and risk for iron overload (ferritin >150 μg/L, all ages).

2 Within a group, values with different superscript letters (a, b, or c) are significantly different, *P* < 0.05 (2-tailed pairwise t-test) and a Bonferroni adjustment was used to correct the p values for the significance test for the multiple comparisons across each socio-demographic characteristic in SF.

3 – percentile value cannot be calculated.

**TABLE 2:** SF (μg/L) concentration thresholds (95% CI in parentheses) identified by hemoglobin and erythrocyte zinc protoporphyrin using restricted cubic spline (RCS) regression with 5 knots in a healthy sample^1^ of United Sates children aged 12–59 mo and nonpregnant women aged 15–49 y participating in the Third National Health and Nutrition Examination Survey (1988–1994)

	Children^2^ (*n* = 2616)	Nonpregnant women^2^ (*n* = 4639)
Hemoglobin (Hb)		
SF corresponding to median Hb plateau point	21.2 (18.5, 26.5)^a^	24.8 (23.4, 26.9)^a^
RCS Model Adjusted R^2^, %	3.5	21.3
		
Erythrocyte zinc protoporphyrin (eZnPP)		
SF corresponding to median eZnPP minima point	18.7 (17.9, 19.7)^a^	22.5 (21.7, 23.3)^b^
RCS Model Adjusted R^2^, %	16.3	23.8

1 Unweighted n and analyses. The following exclusions apply to define a healthy sample: children with infection (white blood cell counts >10.0 × 10^9^/L), nonpregnant women with infection (white blood cell counts >10.0 × 10^9^/L), inflammation (CRP >5.0 mg/L) or possible liver disease (alanine AST >70 U/L or aspartate AST >70 U/L).

2 All plateau and minima estimates and their 95% confidence interval (CI) were obtained from 5000 bootstrap replicates. All CIs have been corrected for bias using the bias corrected acceleration approach (20). Column values with different superscript letters (a or b) are significantly different, p < 0.05. Test for heterogeneity from 2-sided random effect meta-analysis, Cochrane’s Q(1df), p= 0.2654 for children, and p= 0.0163 for nonpregnant women).

**TABLE 3:** SF (μg/L) concentration thresholds identified by mean corpuscular volume, mean corpuscular hemoglobin, and RBC distribution width using restricted cubic spline (RCS) regression with 5 knots in a healthy sample^1^ of United Sates children aged 12–59 mo and nonpregnant women aged 15–49 y participating in the Third National Health and Nutrition Examination Survey (1988–1994)

	Children^2^ (*n* = 2637)	Nonpregnant women^2^ (*n* = 4648)
Mean corpuscular volume (MCV)		
SF corresponding to median MCV plateau point	19.9 (18.5, 22.0)^a^	22.7 (21.6, 23.8)^a^
RCS Model Adjusted R^2^, %	12.0	17.4
MCH		
SF corresponding to median MCH plateau point	19.2 (18.1, 21.3)^a^	22.9 (21.9, 24.1)^a^
RCS Model Adjusted R^2^, %	12.2	19.4
RDW		
SF corresponding to median RDW minima point	19.80 (19.78, 19.83)^a^	23.70 (23.69, 23.73)^a^
RCS Model Adjusted R^2^, %	18.6	23.9

1 Unweighted n and analyses. The following exclusions apply to define a healthy sample: children with infection (white blood cell counts >10.0 × 10^9^/L), nonpregnant women with infection (white blood cell counts >10.0 × 10^9^/L), inflammation (CRP >5.0 mg/L) or possible liver disease (alanine AST >70 U/L or aspartate AST >70 U/L).

2 All plateau and minima estimates and their 95% confidence interval (CI) were obtained from 5000 bootstrap replicates. All CIs have been corrected for bias using the bias corrected acceleration approach (20). Column values with different superscript letters (a or b) are significantly different, p < 0.05. Test for heterogeneity from 2-sided random effect meta-analysis, Cochrane’s Q(2df), p= 0.8589 for children, and p= 0.4221 for nonpregnant women).

## Data Availability

The NHANES data is in public domain and can be downloaded from the NHANES website at https://www.cdc.gov/nchs/nhanes/.

## Abbreviations:

eZnPP: erythrocyte zinc protoporphyrin
Hb: hemoglobin
ID: iron deficiency
MCV: mean corpuscular volume
MEC: Mobile Examination Center
NHANES III: the Third National Health and Nutrition Examination Survey
RCS: restricted cubic spline
SF: serum ferritin
