# Supplementary material for: Comparison of Current World Health Organization Guidelines with Physiologically Based Serum Ferritin Thresholds for Iron Deficiency in Healthy Young Children and Nonpregnant Women Using Data from the Third National Health and Nutrition Examination Survey
Source: J Nutr. Author manuscript; Available in PMC 2023 Sep 1. (PMC10472073; doi:10.1016/j.tjnut.2023.01.035)
Supplement: Supplemental material [file NIHMS1927349-supplement-Supplemental_material.docx]

**Comparison of** **current World Health Organization guidelines with physiologically based serum ferritin thresholds for iron deficiency in healthy young children and non-pregnant women using data from the Third National Health and Nutrition Examination Survey**

Zuguo Mei

**Supplemental Table 1.** Age-specific serum ferritin (SF, µg/L) concentration thresholds (95% confidence interval in parentheses) identified by hemoglobin and erythrocyte zinc protoporphyrin using restricted cubic spline (RCS) regression with 5 knots in a healthy sample^1^ of United Sates children aged 12–59 months participating in the Third National Health and Nutrition Examination Survey (1988–1994)

|  | **12-23 mos**^2^  (n**=**494**)** | **24-59 mos**^2^  (n**=**2122**)** | **p value**^3^  (12-23 vs 24-59 mos) |
| --- | --- | --- | --- |
| **Hemoglobin (Hb)** |  |  |  |
| SF corresponding to median Hb plateau point | 13.1(10.5, 16.4) | 23.5 (19.4, 28.3) | 0.0088 |
| RCS Model Adjusted R^2^, % | 6.4 | 2.2 |  |
|  |  |  |  |
| **Erythrocyte zinc protoporphyrin (eZnPP)** |  |  |  |
| SF corresponding to median eZnPP minima point | 14.5(11.5, 23.8) | 19.4 (18.6, 20.2) | 0.0737 |
| RCS Model Adjusted R^2^, % | 17.3 | 13.5 |  |

^1^Unweighted n and analyses. The following exclusions apply to define a healthy sample: children with infection.

^2^All plateau and minima estimates and their 95% confidence interval (CI) were obtained from 5000 bootstrap replicates. All CIs have been corrected for bias using the bias corrected acceleration (BCa) approach (20).

^3^p-values indicates significant differences in effect sizes at p < 0.05. Test for heterogeneity from 2-sided random effect meta-analysis with Cochrane’s Q at 1df.

**Supplemental Table 2.** Age-specific serum ferritin (SF, µg/L) concentration thresholds (95% confidence interval in parentheses) identified by hemoglobin and erythrocyte zinc protoporphyrin using restricted cubic spline (RCS) regression with 5 knots in a healthy sample^1^ of United Sates non-pregnant women aged 15–49 years participating in the Third National Health and Nutrition Examination Survey (1988–1994)

|  | **15-19 y**^2^  **(n=**810**)** | **20-49 y**^2^  **(n=**3829**)** | **p value**^3^  (15-19 vs 20-49y) |
| --- | --- | --- | --- |
| **Hemoglobin (Hb)** |  |  |  |
| SF corresponding to median Hb plateau point | 21.5 (18.2, 25.0) | 26.4 (24.8, 29.0) | 0.035 |
| RCS Model Adjusted R^2^, % | 16.1 | 22.4 |  |
|  |  |  |  |
| **Erythrocyte zinc protoporphyrin (eZnPP)** |  |  |  |
| SF corresponding to median eZnPP minima point | 18.6 (15.2, 20.1) | 23.6 (22.8, 24.7) | < .0001 |
| RCS Model Adjusted R^2^, % | 25.3 | 23.8 |  |

^1^Unweighted n and analyses.

SF geometric means did not statistically differ between women aged 20-34 y and 35-49 y (Table 1) thus we combined the two groups for the RCS analysis. The following exclusions apply to define a healthy sample: non-pregnant women with infection, inflammation and liver disease.

^2^All plateau and minima estimates and their 95% confidence interval (CI), were obtained from 5000 bootstrap replicates. All CIs have been corrected for bias using the bias corrected acceleration (BCa) approach (20).

^3^p-values indicates significant differences in effect sizes at p < 0.05. Test for heterogeneity from 2-sided random effect meta-analysis with Cochrane’s Q at 1df.
